# A community-based cross-sectional study of the epidemiology of onchocerciasis in unmapped villages for community directed treatment with ivermectin in Jimma Zone, southwestern Ethiopia

**DOI:** 10.1186/s12889-015-1888-x

**Published:** 2015-07-01

**Authors:** Daniel Dana, Serkadis Debalke, Zeleke Mekonnen, Wondwossen Kassahun, Sultan Suleman, Kefelegn Getahun, Delenasaw Yewhalaw

**Affiliations:** Department of Medical Laboratory Sciences and Pathology, College of Health Sciences, Jimma University, Jimma, Ethiopia; Department of Epidemiology and Biostatistics, College of Health Sciences, Jimma University, Jimma, Ethiopia; Department of Pharmacy, College of Health Sciences, Jimma University, Jimma, Ethiopia; Department of Geography and Environmental Science, College of Social Sciences, Jimma University, Jimma, Ethiopia; Department of Medical Laboratory Sciences and Pathology, College of Health Sciences, Jimma University, Jimma, Ethiopia

**Keywords:** Onchocerciasis, Endemicity, Onchocercial skin disease, Microfilaria, Ethiopia

## Abstract

**Background:**

Human onchocerciasis is a neglected tropical parasitic disease caused by *Onchocerca volvulus (O. volvulus)* that may result in devastating skin and eye morbidity. Even though the disease is targeted for elimination, there was little or no information on the level of onchocerciasis endemicity for implementation of community directed treatment with ivermectin (CDTI) in the current study area. Thus, this study aimed at investigating the epidemiology of onchocerciasis and the level of awareness towards the disease among communities living close to CDTI area, Jimma Zone, southwestern Ethiopia.

**Methods:**

A community based cross-sectional study was conducted from April 23 to May 22, 2012. Data on socio-demographic characteristics, knowledge, attitude and practice towards onchocerciasis were collected using semi-structured questionnaires. Clinical examination was undertaken for onchocercal skin diseases by experienced health professionals. Moreover, two skin snip samples were collected from the right and left gluteal folds. Study participants found positive for *O. volvulus* infection during the study were treated individually with standard dose of ivermectin as per WHO guideline.

**Results:**

The overall prevalence of *O. volvulus* infection was 22.5 % while the prevalence of onchocercal skin diseases was 29.8 %. The community microfilarial (mf) load was 5.70 mf per skin snip. Age, sex, educational status, occupation and duration of stay in the villages showed significant association with onchocerciasis (*P* < 0.05). But sex (OR = 0.565, 95 % CI = 0.335, 0.952), educational status (OR = 0.545, 95 % CI = 0.310, 0.958) and duration of stay in the village (OR = 5.933, 95 % CI = 1.017, 34.626) were the independent predictors for *O. volvulus* infection. Three hundred eighty eight (88.2 %) of the study participants reported that they didn’t know about onchocerciasis.

**Conclusions:**

There was moderate prevalence of onchocercal infection and onchocercial skin diseases (OSD) in the study area. Result of this study may suggest that the endemicity level of onchocerciasis in the study area was mesoendemic. Hence, intervention using ivermectin treatment should be implemented to reduce the burden of onchocerciasis. Since the majorities of the population had poor knowledge, attitude and practice towards onchocerciasis, inclusion of health education in the intervention package is crucial.

## Background

Human onchocerciasis is one of the major public health problems among endemic communities. It is a neglected tropical parasitic disease caused by *O. volvulus* and transmitted by the bites of infected black fly (*Simulium* spp*.*)*.* The adult filarial worm lives in subcutaneous nodules and connective tissues over a decade releasing millions of mf that causes OSD and blindness [1].

**Fig. 1:**
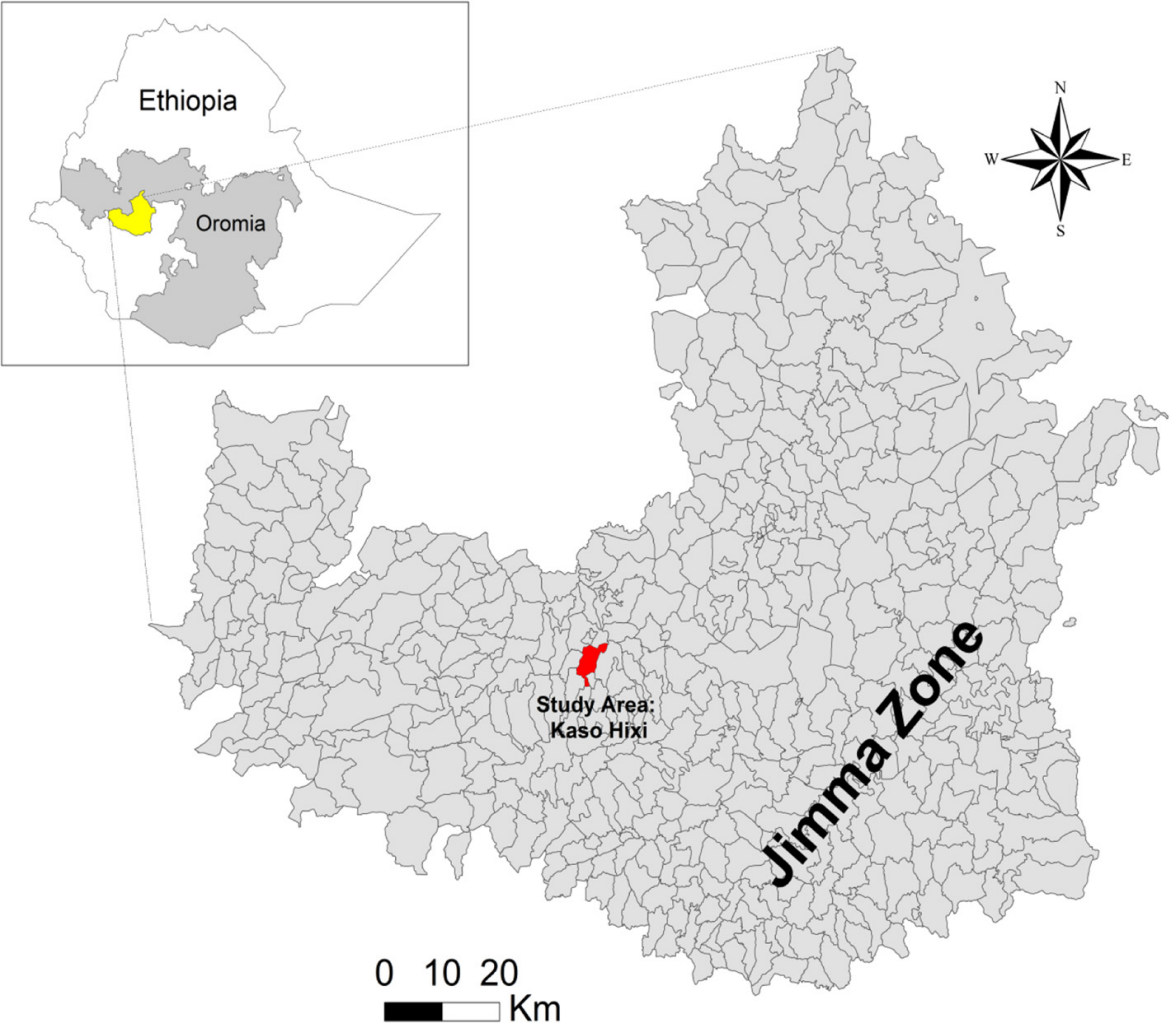
Map of the study area in Jimma Zone, Southwest Ethiopia (own processing, data for administrative boundaries: Ethio-GIS, 2004) [11]

Globally, an estimated 37 million people are infected with *O. volvulus* and 120 million people are at risk [2]. In Ethiopia, an estimated 3 million people are infected and about 10 million people are at risk of infection. It is endemic in Keffa Sheka, Gamo Gofa, Wellega, Gambella, Illubabor and Gondar provinces [3, 4]. The disease has been spreading to previously non endemic regions of Ethiopia due to resettlement of people from endemic areas [5].

An earlier report from Ethiopia indicated that onchocerciasis is responsible for poor school performance and higher dropout rate among infected children. Moreover, there is low productivity, low income, and higher health related costs among infected adults; and extreme forms of social stigmatization, especially among women [6].

Lack of knowledge and misconception towards onchocerciasis could hamper prevention and control efforts. Thus, community knowledge, beliefs and practice towards onchocerciasis is crucial for community participation and acceptance of control strategies [7] and its success. An epidemiological study of onchocerciasis is important to identify high-risk communities to prioritize and implement prevention and control strategies [8]. However, to the best of our knowledge, there was little or no information on onchocerciasis endemicity level and implementation of CDTI in our study area. Currently, the federal minister of health is targeting the elimination of onchocerciasis from some parts of Ethiopia. Nevertheless, there might be a spreading of onchocerciasis from CDTI implemented areas to the neighboring CDTI unimplemented area or there might be a spreading of active infection from neighboring areas without CDTI to CDTI implemented areas. Therefore, those areas unmapped for CDTI may require strong surveillance for the benefit of both CDTI implemented and CDTI unimplemented areas during the elimination era of onchocerciasis in Ethiopia. Since the current study area predominantly coffee growing, there is frequent movement of people from areas without onchocerciasis as a migrant laborer for cultivation of coffee which could intensify onchocerciasis transmission.

Moreover, for successful disease elimination programmes, creating awareness regarding modes of disease transmission, prevention and control in high risk communities is essential. Therefore, the current study was conducted to assess the epidemiology of onchocerciasis and the level of awareness of the communities living in unmapped villages about CDTI in Gomma District, Jimma Zone, southwestern Ethiopia.

## Methods

### Study area

The study was conducted in unmapped villages for CDTI in Kaso Hixi *Kebele*, Gomma District, Jimma Zone, southwestern Ethiopia. The area is located 390 km southwest of the capital, Addis Ababa. Kaso Hixi is a *kebele* (smallest administration unit in Ethiopia) bordered by Choche, Omogobo, Bosooka and Balfo kebeles in North, South, East and West, respectively. The study area lies between latitudes 24°10’96“N and 24°29’63”N, longitude between 86°74’63“E and 86°97’13”E, and an altitude between 1561–1614 masl. Agro ecologically, the study area is classified as *Weina Dega* (Wet Midland) and *Kolla* (Lowland). The rainfall is bimodal in which the short rains are from February to March and the long rainy season lasts from June to September. The mean annual rainfall of the study area is 1524 mm with low variability [9]. According to the lists of population registered in the *kebele* administration, the total population of the study area is about 5289 and the majority of inhabitants are predominantly coffee growers along cattle raising.

### Sample size and sampling technique

Sample size was determined using a single population proportion formula assuming onchocerciasis prevalence rate 17 % from earlier reports [10] with ± 5 % precision which resulted in 440 study participants. The study households were selected using systematic sampling technique from the list of households obtained from the *Kebele* and study participants (age ≥ 15 years) were selected by simple random sampling technique from the selected households.

### Data collection

Data on socio-demographic characteristics, knowledge, attitude and practice towards onchocerciasis, and other associated risk factors were collected using semi-structured questionnaires. Clinical examination was performed by trained and experienced health professionals for the presence of any clinical manifestation of onchocercal skin diseases. Two skin snip samples, one from each side of the gluteal fold were taken using blood lancet and a sterilized razor blade. Each skin snip was weighed using a sensitive analytical balance and placed in coded 96 well micro titer plate wells containing normal saline and kept at room temperature for 24 h for complete emergence of mf. Each plate was examined under a dissecting microscope and the number of mf was counted. For all positive skin snip samples, the number of mf was counted and the average number was recorded. The mean microfilarial load (MFL) was calculated for all positive skin-snip samples. The community microfilarial load (CMFL) was also calculated for all study participants aged ≥ 20 years. All positives skin snip samples were dried, fixed with methanol and stained with Giemsa stain to differentiate the mf of *O. volvulus* from other blood-born mf using morphological characteristics [12].

### Data analysis

Socio-demographic, clinical, and parasitological data were coded, cleaned and analyzed using SPSS software package version 16.0. Bivariate analysis was carried out to determine the association between determinant factors and dependent variables. Variables found to be significant in the bivariate analysis were fitted to multivariate analysis to determine the main predictors. The geometric mean of CMFL was calculated after transforming the count data using log (x + 1) transformation [12] and p-value < 0.05 was considered significant during the analysis.

### Ethical consideration

The study was reviewed and approved by the Research and Ethics Review Committee of Jimma University (Ref.RPCG/141/2012). Official permission for the study was obtained from Jimma Zone and the Gomma District Health Office. Written informed consent was sought from each study participant. Both clinical and laboratory results of the examination were kept confidential and those subjects found positive for onchocerciasis during the study were treated with the standard dose of ivermectin as per the WHO guideline [13].

## Results

### Socio-demographic characteristics

Of the total 440 study participants, 241 (54.7 %) and 199 (45.3 %) were males and females respectively. The mean age of the study participants was 38.3 ± 1.6 years (±1.6 SD) majority being in the age range of 25–34 (25.5 %) years. Two hundred and sixty (59.1 %) of the respondents were farmers, 83 (18.9 %) were housewives and 74 (16.8 %) were students. The majority (50.2 %) of the participants was illiterate (Table 1).

**Table 1:** Socio-demographic characteristics of the study participants, Jimma Zone, southwestern Ethiopia (2012)

Variable	Frequency	(%)
Sex		
Male	241	54.7 %
Female	199	45.3 %
Age		
15-24	89	20.2 %
25-34	112	25.5 %
35-44	85	19.3 %
45-54	65	14.8 %
55-64	49	11.1 %
>64	40	9.1 %
Ethnicity		
Oromo	306	69.5 %
Amhara	50	11.5 %
Dawro	43	9.8 %
Others^a^	41	9.3 %
Religion		
Muslim	332	75.5 %
Orthodox	78	17.7 %
Protestant	30	6.8 %
Educational status		
Illiterate	221	50.2 %
Read and write	73	16.6 %
Elementary	113	25.7 %
Secondary education	33	7.5 %
& above		
Occupations		
Farmers	260	59.1 %
House wives	83	18.9 %
Students	74	16.8 %
Others^b^	23	5.2 %

^a^Others: Kambata, Yem, and Hadiya

^b^Others: Day laborer and Governmental employee

### Prevalence of *O. volvulus* infection and community microfilarial load (CMFL)

The overall prevalence of *O. volvulus* among inhabitants was 22.5 % (99/440). The intensity of microfilaria ranged from 1.00 to 160 per mg of skin snip and the mean intensity was 15.70 mf per mg of skin snip. The overall CMFL was 5.70 mf per skin snip.

### Factors associated onchocerciasis

Age, sex, educational status, occupation and duration of stay in the villages showed significant association with *O. volvulus* infection (*P* < 0.05). However, after adjusting for the effect of other factors: sex (OR = 0.565, 95 % CI = 0.335, 0.952), educational status (OR = 0.545, 95 % CI = 0.310, 0.958) and duration of stay in the village (OR = 5.933, 95 % CI = 1.017, 34.626) were found to be significant independent predictors for *O. volvulus* (Table 2). Goodness of fit of the model was checked by Hosmer and Lemeshow test. Males were twice as likely to have the risk of *O. volvulus* infection as females. Individuals who lived in the village for over 60 years were nearly six times more likely to have the risk of *O. volvulus* infection than those who lived in the village for less than 10 years. Moreover, individuals with education were half as likely to have onchocerciasis as illiterate individuals.

**Table 2:** Results of the bivariate analysis of *O. volvulus* infection and associated risk factors, Jimma Zone, southwestern Ethiopia (2012)

Variables	Skin snip result		OR (95 % CI)	*p*-value
	Positive	Negative		
Sex				
Male	64	177	1	
Female	35	164	0.590 (0.371, 0.938)	0.026*
Age				
15-24	9	80	1	
25-34	16	96	1. 481 (0.621, 3.532)	0.375
35-44	17	68	2.222 (0.931, 5.305)	0.072
45-54	17	48	3.148 (1.301, 7.618)	0.011*
55-64	19	30	5.630 (2.295, 13.809)	<0.001*
>64	21	19	9.825 (3.887, 24.834)	<0.001*
Educational status				
Illiterate	63	158	1	
Read and write	17	56	0.456 (0.277, 0.749)	0.002*
Elementary	13	100	0.813 (0.348, 1.897)	0.632
Secondary education & above	6	27	0.785 (0.314, 1.784)	0.602
Occupation				
Farmers	72	188	1	
Hose wives	19	64	0.775 (0.434, 1.384)	0.389
Students	4	70	0.149 (0.053, 0.424)	<0.001*
Others	4	19	0.550 (0.181, 1.671)	0.292
Distance from River				
<2 km	67	194	1	
≥2 km	32	147	0.630 (0.393, 1.011)	0.056
Duration of stay in the village in years				
1-10	2	13	1	
11-20	5	91	0.357 (0.063, 2.034)	0.246
21-30	28	99	1.838 (0.391, 8.633)	0.440
31-40	20	76	1.711 (0.357, 8.206)	0.502
41-50	14	34	2.676 (0.533, 13.438)	0.232
51-60	18	19	6.158 (1.216, 31.188)	0.028*
>60	12	9	8.667 (1.550, 48.466)	0.014*

*Significant at *p* < 0.05

### Prevalence of onchocercal skin diseases (OSD)

The overall prevalence of OSD was 29.80 %. The prevalence rate of pruritis, palpable nodule, leopard skin and hanging groin were 21.14 %, 3.65 %, 3.41 % and 1.60 %, respectively. All study participants with leopard skin, palpable nodule and hanging groin showed pruritic manifestations.

### Knowledge, attitude and practice of respondents towards onchocerciasis

Of 440 respondents, only 52 (11.8 %) had ever heard of the disease by its local name “Ankoo” while the majority of the respondents 388 (88.2 %) had not heard about the disease previously. Even though 11.8 % of the participants had heard about the disease, they had poor knowledge about its causation, transmission, signs and symptoms of the disease, parts of the body affected, treatability and preventability of the disease (Table 3).

**Table 3:** Knowledge, attitude and practice towards onchocerciasis among respondents, Jimma Zone, southwestern Ethiopia (2012)

Variables	Frequency	Percentage
Ever heard of onchocerciasis		
Yes	52	11.8 %
No	388	88.2 %
Symptoms of the disease		
Skin disfiguring	22	42.3 %
Itching	22	42.3 %
I don’t know	8	15.4 %
Body part affected		
Skin	28	53.8 %
Any part	22	42.3 %
I don’t know	2	3.8 %
Causes of onchocerciasis		
Black fly bite	16	30.8 %
Farm work	6	11.5 %
Contact with infected individual	9	17.3 %
^a^Others	8	15.4 %
I don’t know	13	25 %
Modes of transmission		
Black fly bite	11	21.2 %
Contact with infected individuals	16	30.8 %
^b^Others	12	23.1 %
I don’t know	13	25 %
Most affected group of people		
Adults	1	1.9 %
Young	1	1.9 %
All age groups	26	50 %
I don’t know	24	46.2 %
Onchocerciasis is a serious disease		
Disagree	6	11.5 %
Undecided	1	1.9 %
Agree	45	86.5 %
Disfiguring disease		
Disagree	11	21.2 %
Undecided	3	5.8 %
Agree	38	73.1 %
Onchocerciasis is treatable		
Disagree	2	3.8 %
Undecided	2	3.8 %
Agree	48	92.3 %
Onchocerciasis is curable		
Disagree	14	26.9 %
Undecided	38	73.1 %
Agree	0	0 %
Onchocerciasis is preventable		
Disagree	8	15.4 %
Undecided	24	46.2 %
Agree	20	38.5 %
The role of the community is important		
Disagree	4	7.7 %
Undecided	20	38.5 %
Agree	28	53.8 %
Practice towards preventive measures in community		
Yes	1	1.9 %
No	51	98.1 %
Use health facility for onchocerciasis		
Yes	3	5.8 %
No	49	94.2 %
Use modern medicine for onchocerciasis		
Yes	2	1.9 %
No	50	98.1 %
Wear protective clothes during daily outdoor activities?		
Yes	1	1.9
No	51	98.1
Bathing/washing in rivers		
Yes	52	100 %
No	0	0 %

*OR* = Odds ratio

*AOR* = Adjusted odds ratio

^a^Others: Dirtiness, Excessive sun light

^b^others: Mosquito bite, Sexual intercourse

Attitudes towards the severity of onchocerciasis, disfiguring ability, treatability and possibility of prevention were cited by 45/52 (86.5 %), 38/52 (73.1 %), 48/52 (92.3 %), 20/52 (38.5 %) and 28/52 (53.8 %) of the respondents, respectively. But, none of the participants reported the curability of onchocerciasis. There was no preventive practice reported by respondents towards onchocerciasis (Tables 3).

## Discussion

In the current study, the overall prevalence rate of *O. volvulus* infection was 22.5 %. This finding was lower compared to the results of other studies conducted in Teppi [4, 14] southwestern Ethiopia, around Blue Nile River [15] and West Wellega [16].The difference might be due to long time endemicity of the parasite in the mentioned study area and the higher abundance of vectors in those areas compared to the new emerging foci in the current study. Moreover, Teppi area is known for its large coffee farm and there are many daily laborers who came from different parts of the country and are engaged in outdoor activities and are at the same time exposed to *simulium* bite.

In contrast, the prevalence rate in the current study was slightly higher than the prevalence of onchocerciasis reported from northwestern [17] and southwestern Ethiopia [10, 16, 18] where treatment had already been in place. The present study was undertaken in villages adjacent to CDTI implemented area where the spread of transmission could occur. Moreover, there are numerous fast flowing rivers which provide ideal breeding sites for the vector [9]. On the other hand, the findings of the present study are in agreement with findings from similar study reported from Bure area, southwest Ethiopia [19].

Even though higher prevalence of *O. volvulus* infection and OSD were reported from studies conducted in Nigeria and Sudan [20, 21], there are some studies with similar prevalence of *O. volvulus* infection and higher prevalence of OSD compared to the current study [22]. The observed difference might be due to the difference in parasite strain and vector abundance and infectivity. The prevalence of *O. volvulus* infection was higher in males than females. This is in agreement with the findings reported from several onchocerciasis endemic areas [16–19, 22–23]. This could be attributed to occupational and/or behavioral risk factors. There was a significant association between occupation and prevalence of *O. volvulus* infection in current community. This finding is also in agreement with studies from West Wellega [16] and southwestern Bure [19] from Ethiopia. This could also be attributed to frequent outdoor activities which might expose to continuous *simulium* bite and sustain transmission of the parasite in immunologically naive population.

In addition, duration of stay in the villages was one of the main predictors of onchocercal infection. Consequently, individuals who stayed in the villages for over 60 years were almost six times at higher risk of *O. volvulus* infection than individuals who had stayed in the village between 1 to 10 years. Adults who engaged routinely in outdoor activities were more exposed to the disease .This finding is consistent with another similar study from southwestern Ethiopia [16].

The MFL in the study community ranged from 0 to 160 mf/mg of skin snip while CMFL was 5.70 mf/skin snip. These findings were much lower than other similar studies conducted in Bebeka, southwest Ethiopia [16, 24]. The observed difference might be due to intervention measures taken in the neighboring villages which could influence disease transmission in our study area.

The overall prevalence rate of OSD in the current study was lower than similar studies from West Wellega, Ethiopia [16]. In the later study, hyperendemicity of onchocerciasis and lack of intervention measures could account for the higher OSD.

Over all, the respondents had poor knowledge towards the causation, transmission, treatability, curability, preventability and control of the disease. Moreover, no respondents reported any preventive practice against onchocerciasis. Similarly, poor knowledge and practice towards onchocerciasis was also reported from other places in Ethiopia [7, 5].

### Limitation of the study

Our study design was cross-sectional and it is difficult to make causal inference and to study the transmission dynamics of *O. volvulus* infection. There are many unmapped areas for CDTI in southwestern Ethiopia, but due to resource constraints we only studied few villages and unable to study vector infectivity too.

## Conclusion

The endemicity level of onchocerciasis in the study setting was mesoendemic. Sex, educational status and duration of stay were found to be the main predictor for *O. volvulus* infection. Moreover, there was poor knowledge, attitude and practice towards onchocerciasis in the community. Hence, CDTI and health education should be put in place to reduce the burden of onchocerciasis. Moreover, since the study area was previously unmapped for CDTI, the infection may spread to the neighboring area with CDTI. Therefore, full mapping of endemic areas for CDTI and periodic evaluation of the impact of CDTI in neighboring areas may contribute to elimination of onchocerciasis.
